# Number and Continuous Magnitude Processing Depends on Task Goals and Numerosity Ratio

**DOI:** 10.5334/joc.22

**Published:** 2018-03-23

**Authors:** Tali Leibovich-Raveh, Itamar Stein, Avishai Henik, Moti Salti

**Affiliations:** 1 Department of Mathematics Education, Faculty of Education, The University of Haifa, Haifa, IL; 2 Department of Psychology, Ben-Gurion University of the Negev, Beer-Sheva, IL; 3 The Zlotowski Center for Neuroscience, Ben-Gurion University of the Negev, Beer-Sheva, IL

**Keywords:** Non-symbolic numerosity comparison task, continuous magnitudes, numerosities, sense of number, sense of magnitudes

## Abstract

A large body of evidence shows that when comparing non-symbolic numerosities, performance is influenced by irrelevant continuous magnitudes, such as total surface area, density, etc. In the current work, we ask whether the weights given to numerosity and continuous magnitudes are modulated by top-down and bottom-up factors. With that aim in mind, we asked adult participants to compare two groups of dots. To manipulate task demands, participants reported after every trial either (1) how accurate their response was (emphasizing accuracy) or (2) how fast their response was (emphasizing speed). To manipulate bottom-up factors, the stimuli were presented for 50 ms, 100 ms or 200 ms. Our results revealed (a) that the weights given to numerosity and continuous magnitude ratios were affected by the interaction of top-down and bottom-up manipulations and (b) that under some conditions, using numerosity ratio can reduce efficiency. Accordingly, we suggest that processing magnitudes is not rigid and static but a flexible and adaptive process that allows us to deal with the ever-changing demands of the environment. We also argue that there is not just one answer to the question ‘what do we process when we process magnitudes?’, and future studies should take this flexibility under consideration.

## Introduction

What guides our decision in everyday non-symbolic numerosity comparisons? Imagine being at the grocery store and deciding which line to stand in so that you will have to wait the shortest time possible. What are the factors that you take into account in making such a decision? In contrast to the traditional view that such comparisons will be influenced only (or mostly) by the number of items we see (e.g., Cantlon, Platt, & Brannon, 2009; Dehaene & Changeux, 1993; Feigenson, Dehaene, & Spelke, 2004), recent studies suggested that in these cases, both number and continuous magnitudes are taken into account. In our example, it is not only the number of people waiting in line or the number of items in their shopping cart that you would factor into your decision, but also continuous magnitudes, like the length of the line created by the line of people, or the total surface area and the density of the items in each shopping cart (Gebuis & Reynvoet, 2012a; Leibovich & Henik, 2013; Leibovich, Henik, & Salti, 2015; Leibovich, Vogel, Henik, & Ansari, 2015; Mix, Huttenlocher, & Levine, 2002).

To demonstrate the influence continuous magnitudes has on performance in numerosity comparison tasks, Leibovich and Henik (2014) presented adult participants with groups of between 5–25 dots. Using code created by Gebuis and Reynvoet (2011), the stimuli were designed so that the continuous magnitudes of the dot arrays were not predictive of numerosity. In other words, relying only on continuous magnitudes would have resulted in very low accuracy rates. The ratio between the number of dots in each array, as well as the ratio between 5 different continuous magnitudes, were used as predictors in a stepwise regression [average diameter (because dots differed in size, we computed the average diameter of the dots in an array), total surface area (the sum of surface area for the dots in each array), area extended (also known as convex hull; the smallest contour that included all of the dots, as if an elastic band were wrapped around the dots), density (area extended divided by total surface), and total circumference (the sum of the circumferences of all dots in an array)]. The results revealed that while half of the explained variance in response times (RT) was accounted for by the numerosity ratio, the other half of the variance was explained by different continuous magnitudes ratios, even though these were irrelevant to the task and could not be used as a cue of numerosity. Therefore, the authors suggested that continuous magnitudes are processed automatically, probably due to the natural correlation between number and continuous magnitudes (i.e., usually, more items will take more space, be denser, have more total area, etc., compared with fewer items).

The study mentioned above is only one example representing a large body of evidence demonstrating that when making a non-symbolic numerosity-related decision, not only numerosity but also continuous magnitudes influence performance (Dakin, Tibber, Greenwood, & Morgan, 2011; Tibber, Greenwood, & Dakin, 2012; Hurewitz, Gelman, & Schnitzer, 2006; Leibovich, Henik et al., 2015; Gebuis & Reynvoet, 2013; Smets, Gebuis, & Reynvoet, 2013; Soltész & Szűcs, 2014). In the current study, we aim to take a closer look at the way continuous magnitudes and number interact to influence performance. We will now turn to some examples for questions regarding the interaction of processing numerosity and continuous magnitudes.

### What do we process first – continuous magnitudes or numerosities?

Answers to this question come from Event Related Potential (ERP) studies, and the results are mixed. In an ERP study conducted by Park, DeWind, Woldorff and Brannon (2015), participants passively viewed groups of dots while their brain activity was recorded. The stimuli were constructed so that the number of dots, their size and their density were independent of each other. The results revealed that numerosity was extracted independently of continuous magnitudes 75 ms after the onset of the stimuli.

We suggest that this interpretation should be constrained for several reasons. First, the task involved passive viewing. In passive viewing, participants are not required to make any decision or attend to any particular feature. It may be possible that when task demands changes, more or less weight will be given to continuous magnitudes or numerosities (see Leibovich, Henik, & Salti, 2015). Therefore, the conclusions regarding the timing of numerosity processing should be limited to passive viewing tasks.

Second, in the stimuli used by Park et al. (2015), number and continuous magnitudes correlated (p. 753: “greater IA [dot size] on average yielded smaller N [number of dots]). The level of the correlation between numerosity and continuous magnitudes was not reported. It is possible that this correlation affected the way participants processed the stimuli. In other words, it is not clear whether the same results would be replicated using a set of stimuli that was constructed differently. This stimulus-dependency was demonstrated in another ERP study conducted by Gebuis and Reynvoet (2012b). This study also included a passive viewing task with two different sets of stimuli. In the first experiment, numerosity and continuous magnitudes were correlated. In the second, numerosity and continuous magnitudes were not correlated. In the first experiment, numerosity and continuous magnitudes affected N1 and/or P2 components. In the second experiment, no numerosity-related effects were found. This is an example for how a bottom-up, stimulus-dependent factor can influence magnitude processing even in a passive task.

Soltész and Szűcs (2014) tested the hypothesis that numerosity is a basic visual feature, like shape. If this is true, shapes and numerosity should be processed at about the same time, and relatively early. In an ERP oddball adaptation paradigm, Soltész and Szűcs adapted participants either to a stream of dots or to a specific shape; namely, in one condition, the number of dots changed (i.e., numerosity change condition) and in another condition, the shape of the dot changed (i.e., shape change condition). In the shape change condition, changes in surface and circumference were unavoidable. Thus, the neural change detected in the ERPs can be attributed to changes in either shape or continuous magnitudes. In the shape change condition, there were also significant changes in the N1 and the P2p components that were previously attributed to numerosity. These components were insensitive to change in numerosity. There was a response to the numerical distance between the numerosities in the habituation stream and the deviant condition. This response, however, occurred 600 ms from stimulus onset, which was very late relative to the response to the shape distance effect that occurred after about 150 ms from stimulus onset. Thus, although not designed to directly compare response to continuous magnitudes and numerosity, this study demonstrated that response to continuous magnitudes can occur earlier than to numerosity.

To summarize, the evidence for the question regarding the order of processing numerosity and continuous magnitudes is mixed. The different conditions in which the studies above were conducted (i.e., different stimuli, tasks and task demands) lead us to ask whether such differences can explain the mixed results.

### Is the influence of continuous magnitudes on numerosity processing static or dynamic?

Are the weights given to numerosity and continuous ratio constant or could they change? Some studies have tested a similar question, namely, if and how performance in a numerosity comparison task is affected by factors such as individual differences between participants (e.g., different age and/or culture), differences in stimuli (by using different protocols to construct groups of dots), or differences in the experiment’s procedure (e.g., presenting stimuli for different time durations).

To learn more about how the way in which stimuli are constructed may affect performance, Clayton, Gilmore and Inglis (2015) asked participants to compare the numerosity of two groups of dots. Half of the stimuli were constructed using the Panamath protocol (Halberda, Mazzocco, & Feigenson, 2008) and half with the protocol of Gebuis and Reynvoet (2011). The authors compared the stimuli created by the two protocols and revealed significant differences between the congruity and the ratios of different continuous magnitudes. Accuracy and the size of the congruity effect were affected by the different protocols. Namely, the low-level features of the stimuli influenced performance.

To test how duration of stimuli presentation affected performance, Inglis and Gilmore (2013) presented participants with groups of dots for 16 ms, 300 ms, 600 ms, 1,200 ms or 2,400 ms and asked them to report which group contained more dots. They found that accuracy improved with the time participants took to view the stimuli because the stimuli could be “re-sampled” more times, leading to a more accurate comparison.

Cantrell, Kuwabara and Smith (2015) went even further and compared performance of Japanese and American preschoolers in a match-to-sample paradigm, to test whether cultural differences can also affect the interaction between numerosity and continuous magnitudes. In this study, the authors presented participants with a single group of items (target group) followed by two other groups of items. The participants’ task was to choose which of the two groups of items was most similar to the target group. The children’s decisions were modulated by the number of items in the group, numerosity ratio, continuous magnitude ratio (more specifically, the total area ratio between the target and choice groups), and culture. Namely, children generally relied more on similarity in the number of items when the numerosity of items in a group was small, and when the numerosity ratio was closer to zero (i.e., large difference in numerosity). However, it was found that children’s choices were influenced to a greater extent by similarity of total area when the number of items in a group was large, and when the numerosity ratio was closer to 1 (i.e., small difference in numerosity); Japanese children relied on total area for relatively smaller set sizes more so than American children did. Namely, in this study, performance was not only stimulus-dependent but also culture dependent.

The findings from the studies mentioned above suggest that performance in a numerosity comparison task is further modulated, or configured by many factors. Elaborating and expending on these efforts, we focus on the influence of irrelevant continuous magnitudes on performance in a numerosity comparison task. More specifically, we ask how dynamic the influence of continuous magnitudes on comparison magnitude is, by examining how this effect is modulated by top-down and bottom-up factors.

## The current study

The current study examines whether the weights given to numerosity and continuous magnitude ratios during a non-symbolic numerosity comparison task will be modulated by top-down and bottom-up factors. We hypothesize that magnitude processing is indeed a flexible process, and it is this flexibility that allows us to interact with our environment. In everyday life, we are required to process magnitudes all the time; for example, estimate how long we have left until the next train, which line in the grocery store is longer, or calculate the change given to us in the store. Each task has its own specifications and therefore magnitude processing should be flexible enough to accommodate these different demands.

To that end, we manipulated top-down and bottom-up factors in a non-symbolic numerosity comparison task. The top-down manipulation involved different emphasis on speed or accuracy. Specifically, participants were asked to decide which of two groups of dots contained more dots, as fast and as accurately as they could. Half of the participants were asked to rate, after each trial, how fast they were (on a scale of 6 options; i.e., speed emphasis condition). The other half were asked to rate how confident they were in the accuracy of their response (i.e., the accuracy emphasis condition). We chose to use this method instead of instructing participants at the beginning of the task to be fast or accurate, so we could be as implicit as possible. Specifically, we wanted to avoid a situation where participants were just guessing because speed was more important than accuracy. We also wanted to avoid the opposite condition, when participant become too slow and overcautious, or even count the dots instead of estimating their quantity, because we emphasized accuracy over speed.

The bottom-up factor that was manipulated was the duration in which participants were exposed to the stimuli. The motivation for manipulating the duration of stimuli presentation was inspired by the study of Soltész and Szűcs (2014), who suggested that at the brain level, perceiving changes in the ratio of continuous magnitudes occurs before perceiving changes in numerosity ratio. Therefore, we rationalized that short duration exposures would decrease the impact of numerosity in the comparison task. This factor was previously manipulated by Inglis and Gilmore (2013) and showed a decrease in accuracy with the decrease of exposure duration, albeit without an examination of the influence of continuous magnitude ratio or response times. Inspired by these studies, we decided to manipulate the stimulus presentation time and test whether the relative influence of numerosity and continuous magnitudes would be affected. For all participants, stimuli were presented for either 50 ms, 100 ms or 200 ms. The different exposure durations were mixed within the same block.

If the weight given to different magnitudes is indeed flexible, performance should be modulated by the different manipulation. Previous studies suggested that processing continuous magnitudes is, under some conditions, faster and easier than processing numerosities (e.g., Gebuis, Cohen Kadosh, & Gevers, 2016; Leibovich, Vogel et al., 2015; Soltész & Szűcs, 2014). Therefore, when participants are implicitly pressed for a quick response (like in a speed emphasis condition) and the exposure to the stimuli is brief, participants may rely more on continuous magnitudes, than when emphasis is put on accuracy and the stimuli are exposed for longer time.

To test our hypothesis, and specifically the hierarchical relationship between the different magnitudes in the different duration conditions (speed/accuracy implicit emphasis), we chose to use a multiple stepwise regression. Namely, we used the ratio between the numerosities of the to-be-compared stimuli, as well as the ratio between five different continuous ratios, in a multiple stepwise regression analysis (Leibovich & Henik, 2014) for each condition and duration. We then examined how this modulation affected performance, namely, how relying more on continuous magnitudes would affect RT and accuracy.

### Method

#### Participants

Fifty students (43 females) from Ben-Gurion University of the Negev participated in the experiment for payment. All participants had intact or corrected vision and had not been previously exposed to the current stimuli. Seven participants were left handed and the rest were right handed. The mean age of the participants was 23 years old (*SD* = 1.09 years). Participants were randomly assigned to one of two groups.

#### Stimuli

The stimuli were comprised of two dot arrays (light gray on a black background). We used the same stimuli that were used by Leibovich and Henik (2014). The number of dots varied from 5 to 25 per array. Arrays were paired to create 11 numerosity ratios (smaller numerosity divided by larger numerosity, e.g., 11 vs. 22 dots, which has a ratio of 0.5): 0.2, 0.3, 0.4, 0.5, 0.6, 0.7, 0.75, 0.8, 0.85, 0.9 and 0.95. Complete information about the continuous magnitude ratios can be found online at the following link https://osf.io/gqd6u/. Importantly, the correlations between numerosity and continuous magnitudes were not strong (Table 1), and ranged between –.0039 (non-significant correlation) to .265 (significant correlation), and as such, continuous magnitudes were not a predictive cue of numerosity. The recorded continuous magnitudes were average diameter, total surface area, convex hull (also known as area extended), density, and total circumference. The ratios between these continuous magnitudes for each pair of arrays were recorded. Further details can be found in the study of Leibovich and Henik (2014).

**Table 1:** Correlations between the Different Visual Properties of the Arrays.

Variable	Convex hull	Average diameter	Density	Total surface area	Total circumference	Numerosity

Convex hull	–					
Average diameter	0.08	–				
Density	.139*	.574*	–			
Total surface area	0.064	.657*	.564*	–		
Total circumference	.202*	.567*	.464*	.667*	–	
Numerosity	.219*	–0.039	–.077*	–.213*	.265*	–

*Note. N* = 504; the values represent the Pearson correlation coefficient; * = *p* < .05.

#### Procedure

The study was programmed in OpenSesame (Mathôt, Schreij, & Theeuwes, 2012) version 3.1 for windows. The file is available for use at the following link: https://osf.io/rmbf8/. The screen resolution was 1280 × 1024. The experiment was run on a Windows 7 operating system.

**Figure 1:**
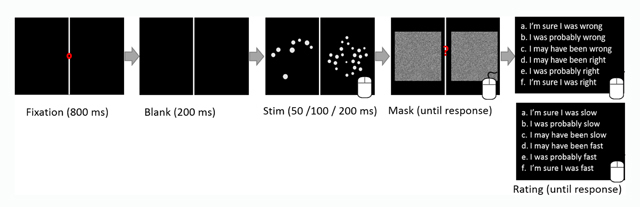
**Procedure.** The procedure was identical in both conditions. The only difference was replacing the words ‘right’ and ‘wrong’ in the accuracy emphasis condition with the words ‘fast’ and ‘slow’, respectively, in the speed emphasis condition. We used letters (a–f) and not numbers in order not to prompt any number representation. The rating scaled appeared in Hebrew since the participants were Hebrew speakers. The speed/accuracy manipulation was made between subjects.

In both conditions, the task was to decide as quickly as possible while avoiding errors, which array contained more dots. Subjects responded by pressing either the left or right key of a computer mouse. Figure 1 depicts the procedure; each trial started with a fixation dot (red dot in the middle of a vertical white line at the center of a black screen) for 800 ms. Two hundred ms after the elimination of the fixation dot, stimuli (dot-arrays) appeared to the left and right of the vertical white line for either 50 ms, 100 ms or 200 ms. After the elimination of the stimuli, a central question mark appeared simultaneously with a mask—two squares with black and white noise (created using OpenSesame’s “Instar noise patch” plugin)—at the location of the previously presented dot-arrays in order to prevent an afterimage. The noise patches were presented until response. A mouse response was possible either during the presentation of the stimuli or during the presentation of the mask. After a response was made, participants were asked to rate either their speed or their accuracy by choosing one of six options: in the accuracy emphasis condition: a) I’m sure I was wrong, b) I was probably wrong, c) I may have been wrong, d) I may have been right, e) I was probably right, f) I’m sure I was right; and in the speed emphasis condition, the words ‘right’ and ‘wrong’ were replaced by the words ‘fast’ and ‘slow’ respectively: a) I’m sure I was slow, b) I was probably slow, c) I may have been slow, d) I may have been fast, e) I was probably fast, f) I’m sure I was fast (Figure 1). The options were presented until response. A response was made by clicking with the mouse on the option the participant felt best described his/her performance. Each participant received 11 practice trials and 396 experimental trials (66 trails per block × 6 blocks).

### Results

#### The influence of emphasis manipulation and stimuli exposure duration on performance

We were interested to see how the different emphasis conditions (emphasis on accuracy or speed) would affect speed, accuracy and efficiency (RT/accuracy), and if this influence would be further modulated by the different stimuli exposure durations. With this aim in mind, we performed 3-way analyses of variance (ANOVAs) for RT, accuracy and efficiency as dependent measures separately.

**Figure 2:**
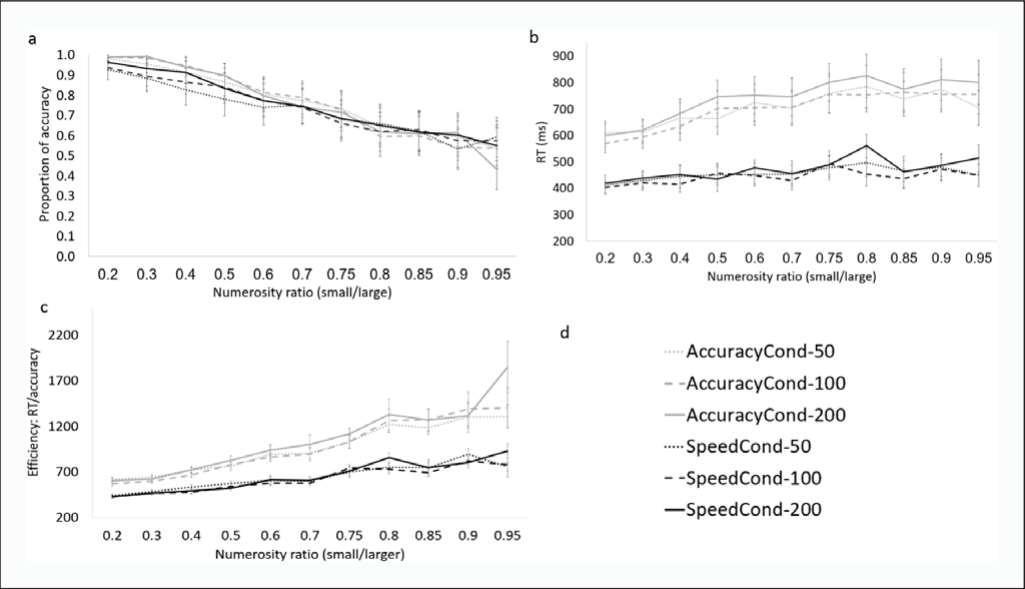
**The influence of emphasis manipulation and stimuli exposure duration on performance. a)** Accuracy as a function of stimuli exposure duration. **b)** RT as a function of stimuli exposure duration. **c)** Efficiency as a function of stimuli exposure duration; note that high efficiency = lower score. **d)** Legend. “AccuracyCond-50” means accuracy emphasis condition and stimuli exposure duration of 50 ms. “AccuracyCond-100” means accuracy emphasis condition and stimuli exposure duration of 100 ms, etc.

**Accuracy.** A 3-way ANOVA with type of emphasis (speed emphasis or accuracy emphasis) as a between-subject independent variable, and with numerosity ratio (divided into bins: 0–0.19, 0.2–0.39, 0.4–0.59, 0.6–0.79 and 0.8–1), and stimuli exposure duration (50 ms/100 ms/200 ms) as within-subject independent variables, and accuracy as a dependent variable, revealed a main effect of numerosity ratio. Namely, accuracy decreased with increase in numerosity ratio, *F* (4, 192) = 693.76, *p* < .001, *η^2^_p_* = .93 (Figure 2a). Numerosity ratio further interacted with type of emphasis, *F* (1, 48) = 5.68, *p* < .001, *η^2^_p_* = .11. The interaction of stimuli exposure duration and numerosity ratio was marginally significant, *F* (8, 384) = 1.85, *p* = .067, *η^2^_p_* = .037. We further explore such interactions in the regression analysis section. The effect of emphasis type was significant, *F* (1, 48) = 5.68, *p* = .064, *η^2^_p_* = .11, suggesting that accuracy affected participants differently when they were asked to emphasize speed or accuracy. However, the effect size in this case was very small—it explained only 11% of the explained variance. There was also no main effect of duration, *F* (2, 96) = 1.68, *p* = .2, *η^2^_p_* = .003, suggesting that accuracy rates for presentation time of 50 ms, 100 ms and 200 ms were not significantly different.

**Response times.** The same ANOVA as described above was performed with RT as the dependent measure. This analysis revealed main effects for emphasis type, stimuli exposure duration and numerosity ratio: participants were significantly faster when speed was emphasized (M speed = 457 ms, M accuracy = 716 ms), *F* (1, 48) = 43.08, *p* < .001, *η^2^_p_* = .47; performance was generally slower for 200 ms (M = 607 ms), than for 100 ms and 50 ms (M = 572 and 580 ms, respectively), *F* (2, 96) = 19.21, *p* < .001, *η^2^_p_* = .29; and RT increased with increase in numerosity ratio, *F* (4, 192) = 53.22, *p* < .001, *η^2^_p_* = .53 (Figure 2b). The interaction between numerosity ratio and stimuli exposure duration was also significant, but the effect size was weak, *F* (8, 384) = 2, *p* = .047, *η^2^_p_* = .04. We discuss this interaction in the regression analysis section.

**Efficiency.** Efficiency score is a measure that combines both speed and accuracy, and aims to summarize the findings of both RT and accuracy analyses. By dividing RT by accuracy we can test how efficient performance is. Fast RT and high accuracy rates indicate high efficiency. In contrast, slow RT and low accuracy indicate low efficiency. Because of the way efficiency is calculated (RT/accuracy), lower scores indicate high performance efficiency whereas higher scores indicate lower performance efficiency. The same ANOVA as described above was performed with efficiency as the dependent measure. The analysis revealed main effects for emphasis type, duration, and numerosity ratio. Performance was more efficient when speed was emphasized (M speed = 642 ms; M accuracy = 1,001), *F* (1, 48) = 44.41, *p* < .001, *η^2^_p_* = .48; efficiency was better when stimuli were presented for 50 ms or 100 ms (M = 807 and 802, respectively) than for 200 ms (M = 856), *F* (2, 96) = 43.44, *p* < .001, *η^2^_p_* = .48; efficiency also decreased with increase in numerosity ratio (Figure 2c), *F* (4, 192) = 159.88, *p* < .001, *η^2^_p_* = .77. The interaction of numerosity ratio and type of emphasis was significant, *F* (4, 192) = 29.86, *p* < .001, *η^2^_p_* = .38. The interaction of numerosity ratio and stimuli presentation and duration was marginally significant, *F* (8, 384) = 1.87, *p* = .064, *η^2^_p_* = .037 (Figure 2c).

#### The influence of numerosity and continuous magnitude ratio is modulated by stimuli exposure time

In most non-symbolic numerosity comparison tasks, only the influence of numerosity ratio is tested. When the influence of continuous magnitudes is tested, it is usually as a group, relating to the influence of congruent or incongruent trials on performance. In the majority of the studies, as is the case in the current work, performance measurements, such as RT and accuracy, are modulated by the ratio between the to-be-compared numerosities (i.e., the ratio effect). This ratio dependency, however, does not exclude the influence of continuous magnitudes, which are usually manipulated in order to reduce their correlation with numerosity, but their influence is not reported. Since our study aimed to ask whether the influence of continuous magnitudes is modulated by top-down and bottom-up factors, we chose to use multiple stepwise regression. This type of analysis allows us to determine which of the different magnitude ratios (numerosity ratio and 5 different continuous magnitude ratios) significantly predicts performance in the different conditions. More details regarding this analysis are provided in the supplementary material.

To estimate the weights given to numerosity ratio and investigate whether these weights were affected by the different experimental conditions, we performed stepwise regression analysis with the numerosity ratio, five continuous ratios, and duration as predictors of accuracy and RT in the different conditions. This analysis allowed us to semi-automatically build a model by successively adding or removing the different predictors (ratio between different magnitudes and the different exposure durations) based solely on the t-statistics of their estimated coefficients.

Commonly, continuous magnitudes are correlated with each other as well as (potentially) with numerosities. This can result in multicollinearity. There are several indications for multicollinearity (Meyers, Gamst, & Guarino, 2006): a correlation value of over .8, VIF (variance inflation factor) of above 10, and tolerance below .01. Since none of these indicators is relevant in our data (see Table 1 and Table S1), we performed a multiple stepwise regression. Only RTs of correct trials were used in the regressions. As in the ANOVA analyses, to have more trials per condition and to achieve more stable results, we grouped the ratios into the following categories: 0–0.19, 0.2–0.39, 0.4–0.59, 0.6–0.79 and 0.8–1.

In the main text, we report the main findings of the regression, namely, the magnitudes that significantly affected performance, arranged from the most to the least influential magnitude. For detailed regression tables and information, please see Supplementary Material).

**Figure 3:**
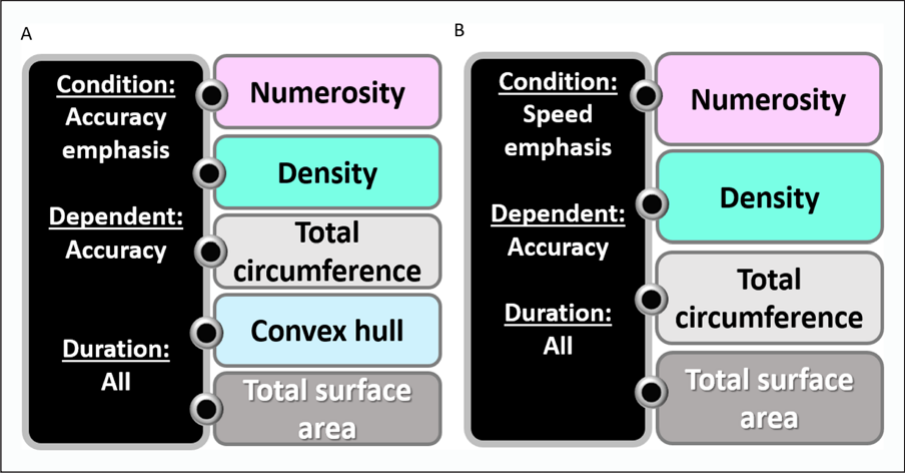
**The hierarchical relationship between different magnitude ratios predicting accuracy in the accuracy and the speed emphasis conditions for all durations. A)** Accuracy emphasis condition. **B)** Speed emphasis condition. The different colors represent different magnitudes. This chart is based on stepwise regression analysis with RT as the dependent measure. The magnitudes are written from top to bottom, representing the most to the least influential predictor. For more details about the regression analysis, see the Supplementary Material.

**Accuracy as a dependent measure. Figure 3** depicts these relationships in the speed emphasis and the accuracy emphasis conditions when the dependent measure was accuracy. The aim of the separate regression analyses was to demonstrate a *qualitative* difference between magnitudes that influence performance in each of the different experimental conditions. As can be seen in Figure 3, for both the accuracy and speed emphasis conditions, numerosity ratio was the most influential magnitude, followed by density, total circumference and total surface area ratios. Convex hull was found to significantly predict accuracy only in the accuracy emphasis condition. Duration did not significantly predict accuracy in either condition.

**Figure 4:**
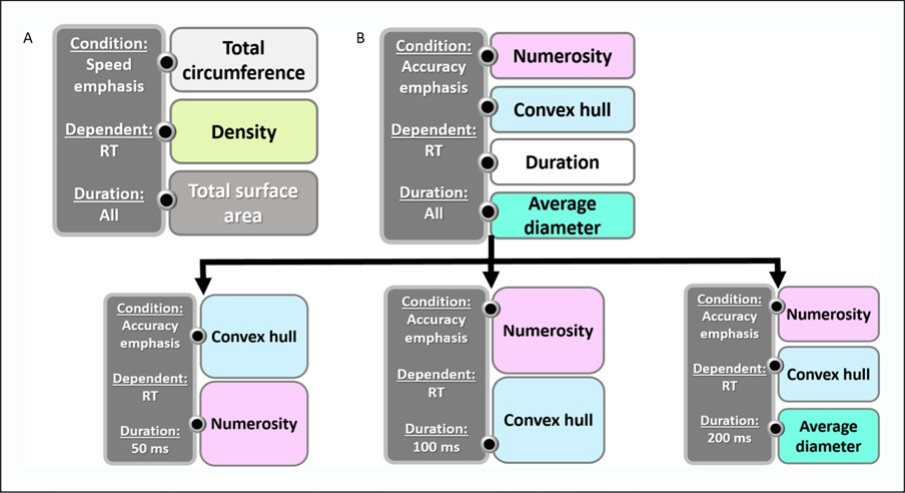
**The hierarchical relationship between different magnitude ratios predicting accuracy in the speed and the accuracy emphasis conditions. A)** Speed emphasis condition. **B)** Accuracy emphasis condition. Since duration predicted RT, a separate regression analysis was performed for every duration. The different colors represent different magnitudes. This chart is based on stepwise regression analysis with RT as the dependent measure. The magnitudes are written from top to bottom representing the most to least influential predictor. For more details about the regression analysis, see the Supplementary Material.

**RT as dependent measure. Figure 4** depicts the relationships in the speed emphasis and the accuracy emphasis conditions when the dependent measure was RT. As can be seen in Figure 4A, there were only three magnitudes that their ratios significantly predicted RT across different durations: total circumference, density and total surface area. Importantly, when considering numerosity ratio as the only magnitude (in an ANOVA analysis), numerosity ratio was found to affect RT. However, it seems that when considering other magnitudes as well, the effect of numerosity became only marginally significant (*t* = 1.84, *p* = .067) and was therefore excluded from the analysis.

In the accuracy emphasis condition, however, a different picture emerges (Figure 4B). Specifically, numerosity ratio was the most influential predictor of RT, followed by convex hull, stimuli exposure, duration and average diameter ratios. Since duration affected RT, we performed a separate regression for each duration. For a duration of 50 ms, the most influential magnitude was convex hull ratio, followed by numerosity ratio. For a duration of 100 ms, however, the most influential magnitude was numerosity ratio, followed by convex hull ratio. For a duration of 200 ms, numerosity ratio, followed by convex hull and average diameter ratios predicted RT.

### Discussion

Given the natural correlation between non-symbolic numerosities and continuous magnitudes, and recent evidence for the important role continuous magnitudes play in non-symbolic numerosity processing, we asked whether the influence of continuous magnitudes is static (consistent and fixed across different conditions) or dynamic. Our results provided evidence for the dynamic nature of the magnitude comparison process. Participants’ reliance on numerosity or on continuous magnitudes was modulated by different top-down and bottom-up factors. Namely, the hierarchy of the influence of numerosity and continuous magnitudes was modulated by task instructions, task difficulty and exposure duration.

#### The influence of numerosity ratio and continuous ratios is modulated by stimuli presentation time

We found that participants’ RT was affected differently by the different numerosity ratios and continuous magnitude ratios in the different durations. More specifically, in the accuracy emphasis condition when stimuli were presented for 50 ms, convex hull ratio was the most influential predictor of RT; in the speed emphasis condition, the influence of numerosity ratio on RT was not significant for either presentation time. Note that not only the influence of numerosity ratio changed, but also the hierarchy of the influence of different continuous magnitudes. Just like task demands can result in different strategies, making numerosity ratio more or less influential, they can do the same for different continuous magnitude ratios. In other words, just like the different task demands affect processing of numerosities, they affect processing of continuous magnitudes.

We would like to argue that this pattern of results is consistent with ERP findings (Soltész & Szűcs, 2014), suggesting that detecting change in continuous magnitudes ratio occurs faster than detecting a change in numerosity ratio. The current work adds to the study of Soltész and Szűcs by using a single numerosity comparison task to demonstrate that under some conditions, namely, fast stimulus presentation and implicit encoruagement to perform faster, continuous magnitude ratios influence RT more than numerosity ratio even when continuous magnitudes are irrelevant to the task and are not a consistent predictor of numerosity.

The study by Park et al. (2015), discussed in the introduction, suggested that numerosities are processed earlier than continuous magnitudes. However, as published in the supplementary material of a recent study applying the method of DeWind et al. (Starr, DeWind, & Brannon, 2017), the correlation between numerosity ratio and item area, total surface area and sparsity randged between 0.66–0.68. Therefore, participants were able to base their response on any of these continuous magnitudes and still have high accurcy (at least above chance level). As a result, it is difficult, if not impossible, to dissociate the influence of numerosity ratio from that of continuous magnitude ratios on performance in this study. In our study, there was also a significant correlation between some continuous magnitudes and nuemrosity (see Table 1). However, these correlations were lower compared with the correlations of Starr et al. (2017) and ranged between 0.08–0.27. Yet, we do not claim that our design is able to completely separate the influence of numerosity and continuous magnitudes – we can only compare this influnece under different conditions.

It is important to mention that what we measured is not whether numerosity by itself influenced performance. Numerosity is the relevant dimension and, given the nature of the stimuli (the low correlation with continuous mangitudes), participanats must have attended to the number of dots in every group in order for performance to be as high as it was. What the regression and the ANOVAs tested was whether the *ratio* between the numerosities affected perfomrance. According to Banks (1977), it is possible that number will influence performance and still the numerical distance will not affect RT. Banks, however, demosntrated this possibilty with single-digit symbolic number comparisons, and more research is needed to apply the same conclusions to non-symbolic numerosities.

#### Stimuli exposure duration does not affect accuracy

Our work compliments the findings of Inglis and Gilmore (2013). In their study, the authors exposed participants to two groups of dots for 16 ms, 300 ms, 600 ms, 1,200 ms or 2,400 ms and asked them to report which group contained more dots. The authors reported that accuracy increased with presentation time, and suggested a process of re-sampling that improves accuracy. In the current work, we focused on brief exposures in order to test how numbers are compared during brief presentations, in order to unravel a more primary and crude mechanism. Under these conditions, exposure time did not affect accuracy. We suggest that between 50 ms–200 ms, re-sampling may not be possible, or efficient enough to improve accuracy. In this time window, we found that when accuracy was emphasized (compared to when speed was emphasized), different magnitude ratios accounted for variability in RTs for the different durations. We elaborate on these findings below.

#### The influence of top-down and bottom-up processes in magnitude comparison tasks

When speed was emphasized, we found only about a 10% drop in accuracy rate (see Figure 2a). The effect size was small. In the same condition, however, participants performed 36% faster with a minimal effect on accuracy. Since performance was much faster and only slightly less accurate, we can say that in the speed emphasis condition, performance was more efficient. This was also demonstrated by the efficiency analysis. The subsequent regression analyses revealed that in the speed emphasis condition, RT was not significantly affected by numerosity ratio, but only by continuous magnitude ratios. On the other hand, when accuracy was emphasized, RT was first affected by numerosity ratio and then by continuous magnitudes (Figure 4). Combining these findings suggests that efficiency was higher when speed was emphasized and numerosity ratio did not affect RTs.

Since for the same stimuli, numerosity ratio affected RTs in the accuracy emphasis condition but not in the speed emphasis condition, we suggest that processing numerosity ratio is strategy-dependent. We argue that this occurs because for short durations of stimuli presentation (up to 200 ms), an elaborated processing of numerosity (resulting in numerosity ratio affecting RT) is not the optimal strategy for solving the task; relying more on continuous magnitudes provided only slightly lower accuracy rates in a much shorter response time. It is plausible, however, that with longer durations of stimuli presentation, like the one used by Inglis and Gilmore (2013), relying on numerosity ratio would increase efficiency. Our results also raise the possibility that re-sampling can be qualitatively different depending on task demands. In other words, is it possible that in every re-sample, we attend to different magnitudes, thereby improving the efficiency of the re-sampling process? This question remains open for further studies.

#### Accuracy and RT as complimentary measures of performance

Since accuracy explains more of the variance than RT, some studies have tested only accuracy in a magnitude comparisons task (e.g., Gebuis & Reynvoet, 2012a; Inglis & Gilmore, 2013). In the current work, we measured both accuracy and RT and found that regression analysis with accuracy as the dependent measure yielded different results from those of regression with RT as a dependent measure.

We argue that in numerosity comparison tasks, accuracy and RT are complimentary measures of performance; accuracy represents the end-result of a decision process (e.g., choosing the left or the right group of dots), while RT reflects the cognitive processes occurring during the decision process. For example, in different trials, participants can reach a correct decision, however, reaching that decision can differ according to the experimental conditions, revealing additional information about the processes underlying the decision.

The explained variance of accuracy is much higher than that of RT (R^2^ = .47 for accuracy compared with R^2^ = .26 for RT; see Supplementary Material), making accuracy a more attractive measure of performance. However, we would like to argue that RT is an important measure as well, since it explains a different part of the cognitive process. In the current study, for example, we found that when speed was emphasized, numerosity ratio was a significant predictor of accuracy, but not of RT. This means that while numerosity ratio affected the end result (whether the answer was correct or not), it failed to significantly affect the time it took to reach this decision. This is an example for the usefulness of measuring both accuracy and RT in magnitude comparison tasks.

#### Processing magnitudes is a flexible and adaptive process

The current work demonstrated that processing magnitudes was more flexible and adaptive than we thought in the past. Additional evidence for this flexibility comes from imaging and behavioral work. For example, in an fMRI study, Leibovich, Henik and Salti (2015) asked adults to compare either the numerosity or the total surface area of 2–4 dots. Half of the participants started with the numerosity task and half with the area task. Behaviorally, continuous magnitudes affected performance in the two groups, resulting in a congruity effect. Namely, in the numerosity task, when continuous magnitudes were incongruent with number (i.e., fewer dots had greater total surface area than more dots did), RT increased and accuracy decreased compared with congruent trials (i.e., fewer dots had smaller total surface area than more dots did). However, in the area task, the congruity effect was significant only for the group that started with the numerosity task. Namely, the automatic processing of numerosities was modulated by task context. At the brain level, there was a main effect of order, suggesting the possibility that the order of the task affected the strategy chosen by the participants in the two groups.

Is such flexibility specific to comparison tasks? A recent line mapping task reported similar flexibility (Leibovich, Al-Rubaiey Kadhim, & Ansari, 2017). In this study, participants were briefly (200 ms) exposed to 2–8 dots and were asked to map the stimuli onto a line between 0 and 10 dots according to their numerosity. Continuous magnitudes affected performance only when accurate estimation was not possible, that is, for numerosities above the subitizing range. Moreover, the influence of continuous magnitudes on numerosity estimation is qualitatively different from its influence in a comparison task; in estimation tasks, the numerosity of small-size objects is over-estimated while the numerosity of large-size objects is under-estimated. This is only one example of how much the nature of the task and the stimuli can affect the way numerosity and continuous magnitudes interact.

A flexible and adaptive magnitude system makes sense in light of the different and ever changing demands of the environment. In our daily lives, we often face the same stimuli, but under different top-down or bottom-up conditions. For example, sometimes you only have a brief moment to make a numerical judgment, like choosing the lane with less cars while driving. Sometimes, there is more time to make a decision, like when choosing the part of the parking lot where more parking space is available. In both these cases, an estimate of the number of cars should be enough to reach a decision, and using continuous magnitudes would be appropriate. However, sometimes task demands require one to ignore some magnitudes and be more attentive to others. For example, when following a recipe that requires exactly three eggs, one would be wise not to estimate the number of eggs by their continuous properties, but to accurately select exactly three eggs. Such variety of demands requires dynamic and adaptive magnitude processing.

Recognizing the adaptive nature of magnitude processing is highly important and relevant for the field of numerical cognition, and especially regarding the question of what do we process when we process magnitudes. Accumulating evidence demonstrates that this question is too broad, since the magnitudes we process and how we process them is highly dynamic and changes depending on the component of the stimuli, task demands, task goals, culture, age, and many other factors (e.g., Cantrell, Boyer, Cordes, & Smith, 2015; Cantrell & Smith, 2013; Gilmore et al., 2016; Leibovich & Henik, 2014; Leibovich, Henik et al., 2015; Salti, Katzin, Katzin, Leibovich, & Henik, 2017). This dynamic nature of magnitude processing raises many new and important questions, such as the flexibility of magnitude processing at different ages, which factors can interfere or improve magnitude processing flexibility, and so forth.

## Summary and Conclusion

In the current work, we looked deeper into the nature of the influence of continuous magnitudes on non-symbolic numerosity comparisons. Our results are in line with previous studies arguing that in order to make a numerical judgment, different weights are given to different magnitudes (Gevers, Cohen Kadosh, & Gebuis, 2016; Leibovich & Ansari, 2016; Leibovich & Henik, 2014; Leibovich, Katzin, Harel, & Henik, 2017). Here, however, we took a step forward and suggested that such weights are not static, but dynamic, and are applied flexibly. The weights given to different magnitudes depend not only on the stimuli, but also on task demands and on the relationship between different top-down and bottom-up factors. Processing magnitudes is adaptive, allowing us to successfully deal with an environment with ever changing demands. Accordingly, we suggest that there is not just one answer to the question ‘what do we process when we process magnitudes?’ Designing studies with the notion of flexibility in mind can shed new light on the processing of magnitudes and their possible role as the building blocks of mathematics.

## Data Accessibility Statement

The data of this ms is available online in the link: https://osf.io/rmbf8/.

## Additional File

The Additional file for this article can be found as follows:

10.5334/joc.22.s1 Supplementary material. Elaborated regression analysis.
