# Supplementary material for: Number and Continuous Magnitude Processing Depends on Task Goals and Numerosity Ratio
Source: J Cogn. 2018 Mar 23;1(1):19. doi: 10.5334/joc.22 (PMC6634598; doi:10.5334/joc.22)
Supplement: Supplementary material. — Elaborated regression analysis. [file joc-1-1-22-s1.pdf]

Supplementary Material

Accuracy emphasis condition

RT as a dependent measure

In step 1 of the analysis, numerosity ratio was entered into the regression equation and was significantly related to RT, F (1, 370) = 69.19, p < .001. The correlation coefficient was .4, indicating approximately 16% of the variance in RT could be accounted for by the numerosity ratio. In the next steps, in addition to numerosity ratio, convex hull ratio, duration and average diameter ratio were entered into the regression equation. In the final regression step, the correlation coefficient was .51, indicating 26% of the variance of the RT could be accounted for by the duration and the mentioned numerosity and continuous ratios (see Table S2).

Since duration affected RTs, we conducted the same regression but separately for each duration. For durations of 50 ms, convex hull ratio was the first to enter, F (1, 122) = 9.95, p = .002. The correlation coefficient was .28, indicating approximately 7.5% of the variance in RT could be accounted for by convex-hull ratio. In the next and final step, in addition to convex hull ratio, numerical ratio was entered into the equation. In this final regression step, the correlation coefficient was .35, indicating 12% of the variance of the RT could be accounted for by the mentioned continuous ratios. For durations of 100 ms, numerosity was the first to enter into the regression, F (1, 122) = 33.85, p < .001. The correlation coefficient was .47, indicating approximately 22% of the variance in RT could be accounted for by the numerosity ratio at an exposure duration of 100 ms. In the second and final step, convex hull ratio was entered into the equation. The correlation coefficient was .58, indicating approximately 34% of the variance in RT could be accounted for by numerosity and convex hull ratios at exposure durations of 100 ms (see Table S3). For durations of 200 ms, numerosity ratio was the first to enter into the regression, F (1, 122) = 35.23, p < .001. The correlation coefficient was .48, indicating approximately 22% of the variance in RT could be accounted for by numerosity ratio at exposure durations of 200 ms. In the final step, convex hull and average diameter ratios were entered into the equation. The correlation coefficient was .55, indicating approximately 31% of the variance in RT could be accounted for by numerosity and convex hull ratios at exposure durations of 100 ms (see Table S3).

Accuracy as a dependent measure

In step 1 of the analysis, numerosity ratio was entered into the regression equation and was significantly related to accuracy, F (1, 500) = 334.34, p < .001. The correlation coefficient was -.63, indicating approximately 40%

of the variance in accuracy rates could be accounted for by the numerosity ratio. In the next steps, in addition to numerosity ratio, density, total circumference, convex hull and total surface area ratios were entered into the regression equation. In this final regression step, the correlation coefficient was .69, indicating 47% of the variance of the accuracy rates could be accounted for by the mentioned numerosity and continuous ratios (see Table S4). Note that duration was not entered into the regression.

Speed emphasis condition

RT as a dependent measure

In step 1 of the analysis, total circumference ratio was entered into the regression equation and was significantly related to RT, F (1, 371) = 45.9, $p < .001$. The correlation coefficient was .33, indicating approximately 11% of the variance in RT could be accounted for by numerosity ratio. In the next steps, in addition to total circumference ratio, density and total surface ratio were entered into the regression equation. In the final regression step, the correlation coefficient was .38, indicating 15% of the variance of the RT could be accounted for by the mentioned continuous ratios (see Table S5). Note that neither numerosity ratio nor duration were entered into the regression equation.

Accuracy as a dependent measure

In step 1 of the analysis, numerosity ratio was entered into the regression equation and was significantly related to accuracy, F (1, 500) = 234.68, $p < .001$. The correlation coefficient was -.57, indicating approximately 32% of the variance in accuracy could be accounted for by numerosity ratio. In the next steps, in addition to numerosity ratio, density, total circumference and total surface ratios were entered into the regression equation. In the final regression step, the correlation coefficient was .66, indicating 44% of the variance of the accuracy rates could be accounted for by the mentioned magnitude ratios (see Table S6).

Table S1

Collinearity Statistics

Regression name	Accuracy		RT	
Variable	Tolerance	VIF	Tolerance	VIF
Convex hull	0.917	1.09	---	---
Average diameter	0.683	1.46	---	---

Density	0.889	1.13	0.998	1
Total circumference	---	---	---	---
Total surface area	0.248	4.03	---	---
Numerosity	0.267	3.75	0.998	1

Note. VIF = variance inflation factor.

Table S2

Summary of Multiple Stepwise Regression Analysis for Variables Predicting RT in the Accuracy Emphasis Condition

	RT - across durations			
Step and variable	B ± SE	β	R^2	Change in R^2
Step 1			.158*	
Numerosity	254.36±30.58	.4*		
Step 2			.222*	.064*
Numerosity	219.16±30.1	.342*		
Convex hull	138.31±24.91	.261*		
Step 3			.246*	.024*
Numerosity	219.16±29.67	.342*		
Convex hull	138.31±24.56	.261*		
Duration	0.31±0.09	.155*		
Step 4			.259*	.013*
Numerosity	209.16±29.72	.326*		
Convex hull	142.15±24.43	.268*		
Duration	0.309±0.09	.155*		
Average diameter	-123.37±49.15	-.114*		

* $p < .05$

Table S3

Summary of Multiple Stepwise Regression Analysis for Variables Predicting RT in the Accuracy Emphasis Condition by Duration

Step and variable	B ± SE	β	R^2	Change in R^2
RT - for duration of 50 ms				
Step 1			.075*	
Convex hull	134.44±42.6	.275*		
Step 2			.119*	.044*
Convex hull	112.31±42.61	.229*		
Numerosity	126.87±51.59	.215*		
RT - for duration of 100 ms				
Step 1			.217*	
Numerosity	264.58±45.46	.466*		
Step 2			.337*	.12*
Numerosity	222.24±42.98	.392*		
Convex hull	166.31±35.58	.354*		
RT - for duration of 200 ms				
Step 1			.224*	
Numerosity	343.02±57.79	.475*		
Step 2			.273*	.049*
Numerosity	308.29±57.42	.146*		
Convex hull	136.44±47.55	.146*		
Step 3			.307*	.034*
Numerosity	290.03±56.86	.429*		
Convex hull	143.47±46.74	.429*		
Average diameter	-225.31±94.02	.211*		

* $p < .05$

Table S4

Summary of Multiple Stepwise Regression Analysis for Variables Predicting Accuracy in the Accuracy Emphasis Condition

	Accuracy - across durations			
Step and variable	B ± SE	β	R^2	Change in R^2
Step 1			.401*	
Numerosity	-.566±.031	.-.633*		
Step 2			.423*	.021*
Numerosity	-.58±.031	-.649*		
Density	-.115±.027	-.147*		
Step 3			.449*	.026*
Numerosity	-.63±.032	-.704*		
Density	-.184±.03	-.235*		
Total circumference	-.148±.03	-.191*		
Step 4			.466*	.017*
Numerosity	-.601±.032	-.672*		
Density	-.167±.03	-.213*		
Total circumference	-.158±.03	-.202*		
Convex hull	-.129±.032	-.138*		
Step 5			.471*	.005*
Numerosity	-.564±.036	-.631*		
Density	-.189±.031	-.242*		
Total circumference	-.102±.04	-.131*		
Convex hull	-.127±.032	-.137*		
Total surface area	-.081±.039	-.113*		

* $p < .05$

Table S5

Summary of Multiple Stepwise Regression Analysis for Variables Predicting RT in the Speed Emphasis Condition

	RT - across durations			
Step and variable	B ± SE	β	R^2	Change in R^2
Step 1			.11*	
Total circumference	50.49±7.45	.505*		
Step 2			.126*	.016*
Total circumference	44.77±7.75	.475*		
Density	20.8±8.1	.137*		
Step 3			.148*	.022*
Total circumference	55.98±8.46	.475*		
Density	31.18±8.69	.137*		
Total surface area	-27.94±9.11	.117*		

* $p < .05$

Table S6

Summary of Multiple Stepwise Regression Analysis for Variables Predicting Accuracy in the Speed Emphasis Condition

	Accuracy - across durations			
Step and variable	B ± SE	β	R^2	Change in R^2
Step 1			.32*	
Numerosity	-.52±.022	-.566*		
Step 2			.414*	.094*
Numerosity	-.549±.032	-.598*		
Density	-.247±.028	-.308*		
Step 3			.422*	.008*
Numerosity	-.521±.033	-.567*		
Density	-.208±.031	-.26*		
Total circumference	-.084±.032	-.105*		
Step 4			.437*	.015*
Numerosity	-.453±.038	-.493*		
Density	-.249±.033	-.31*		
Total circumference	-.185±.042	-.232*		
Total surface area	-.148±.041	-.201*		

* $p < .05$
